# Microstructure Refinement in W-Y_2_O_3_ Alloy Fabricated by Wet Chemical Method with Surfactant Addition and Subsequent Spark Plasma Sintering

**DOI:** 10.1038/s41598-017-06437-z

**Published:** 2017-07-20

**Authors:** Zhi Dong, Nan Liu, Zongqing Ma, Chenxi Liu, Qianying Guo, Zeid Abdullah Alothman, Yusuke Yamauchi, Md. Shahriar A. Hossain, Yongchang Liu

**Affiliations:** 1 Tianjin Key Laboratory of Composite and Functional Materials, School of Materials Science and Engineering, Tianjin University, Tianjin, 300072 China; 2 Advanced Materials Research Chair, Chemistry Department, College of Science, King Saud University, Riyadh, 11451 Saudi Arabia; 3 International Center for Materials Nanoarchitectonics (MANA), National Institute for Materials Science (NIMS), 1-1 Namiki, Tsukuba, Ibaraki 305-0044 Japan; 4 Australian Institute for Innovative Materials (AIIM), University of Wollongong, Squires Way, North Wollongong, NSW 2500 Australia

## Abstract

With the aim of preparing high performance oxide-dispersion-strengthened tungsten based alloys by powder metallurgy, the W-Y_2_O_3_ composite nanopowder precursor was fabricated by an improved wet chemical method with anion surfactant sodium dodecyl sulfate (SDS) addition. It is found that the employment of SDS can dramatically decrease W grain size (about 40 nm) and improve the size uniformity. What’s more, SDS addition can also remarkably improve the uniform dispersion of Y_2_O_3_ particles during the synthesis process. For the alloy whose powder precursor was fabricated by traditional wet chemical method without SDS addition, only a few Y_2_O_3_ dispersoids with size of approximate 10–50 nm distribute unevenly within tungsten grains. Nevertheless, for the sintered alloy whose powder precursor was produced by improved wet chemical method, the Y_2_O_3_ dispersoids (about 2–10 nm in size) with near spherical shape are dispersed well within tungsten grains. Additionally, compared with the former, the alloy possesses smaller grain size (approximate 700 nm) and higher relative density (99.00%). And a Vickers microhardness value up to 600 Hv was also obtained for this alloy. Based on these results, the employment of SDS in traditional wet chemical method is a feasible way to fabricate high performance yttria-dispersion-strengthened tungsten based alloys.

## Introduction

With many distinctive physical properties such as high melting point, low vapor pressure, good thermal conductivity, high strength at elevated temperatures, low coefficient of thermal expansion, low sputtering rate and low tritium inventory^1–6^, tungsten and tungsten-base alloys have been extensively applied in various fields, such as mechanical engineering^7^, electronics^8^, aerospace^9^ and fusion systems. However, intrinsic drawbacks, relatively high ductile-brittle transition temperature (DBTT) and serious embrittlement^10–12^, always limit their wide application.

It is well accepted that the brittleness of tungsten-base alloys is extremely sensitive to their microstructure including tungsten grain size, the uniformity of structure, porosity and grain boundary segregation of impurity elements. Above all, the ultrafine grained structure which depends on initial tungsten grain size, sintering process and the solubility of tungsten in bonding phase can significantly improve the strength and hardness of tungsten-base alloys^11, 12^. Adopting ultrafine composite powder precursor and rapid sintering rate is a highly effective way to obtain fine-grained tungsten-base alloys. Besides, a uniform distribution of bonding phase in tungsten matrix can enhance integrated mechanical properties of the materials, hence is also a key parameter for obtaining high-performance tungsten-base alloys^13^. On the other hand, the non-homogeneous structure is always the zone where stress concentration can occur easily, causing a reduction of composite material in strength. Moreover, it is a high degree of crystal disorder and impurity (e.g., N and O) segregation at grain boundaries that decrease the grain boundary cohesion, which is generally considered the predominant reason why tungsten-base alloys possess inadequate ductility^14^. In addition, high porosity is also detrimental to the performance of the materials. Thus, it is essential to prepare tungsten-base alloys with ultrafine grains, uniformly distributed dispersion phase particles and high relative density to enhance the comprehensive mechanical properties of the materials.

In recent years, lots of studies have reported that rare earth oxides including Y_2_O_3_, La_2_O_3_ and Ce_2_O_3_ have a positive effect on improving microstructure and mechanical properties of tungsten-based alloys. The machinability and recrystallization temperature of the materials are enhanced significantly because of the addition of these oxides^15, 16^. Besides, the oxides distributed at grain boundaries, as the obstacles for grain boundaries migration, can inhibit the growth of tungsten grains during sintering^17^. Finally, due to strong rare-earth-oxygen interactions, the rare earth oxides can gather impurity elements and purify grain boundaries^18^. In recent years, the oxide-doped tungsten composite powder precursors are mainly fabricated by mechanical alloying and emerging wet chemical method. However, it must be mentioned that the grain size of composite powder precursor is relatively large and the size distribution is quite wide^19, 20^. There is no denying that this kind of powder precursor will cause abnormal grain growth during subsequent SPS. Besides, the oxide particles in powder precursor distribute inhomogeneously and tend to agglomerate at grain boundaries to some extent, greatly weakening the improvement effect of rare earth oxides on the properties of tungsten-base alloys, especially the DBTT and recrystallization temperature. There is thereby an urgent need but it is still a significant challenge to prepare oxide-doped tungsten composite powder precursors consisted of uniformly ultrafine tungsten grains and homogeneously dispersed oxide grains. In previous papers, polyvinyl pyrrolidone (PVP) and polyethylene glycol (PEG) were added to prepare W-Y_2_O_3_ powder precursor and the tungsten (W) grain size is improved greatly due to the steric hindrance effect of organic molecules^21, 22^. However, the size and uniform distribution of Y_2_O_3_ particles within the W grains in the sintered W-Y_2_O_3_ alloy still need to be improved.

In present work, the W-Y_2_O_3_ composite powder precursor was synthesized by an improved wet chemical method where ultrasonic process and anion surfactant sodium dodecyl sulfate (SDS) were innovatively introduced. For comparison, W-Y_2_O_3_ composite powder precursor was also fabricated by traditional wet chemical method. It is found that the high-quality W-Y_2_O_3_ composite powder consisted of uniform nano W grains (about 40 nm) and homogeneously dispersed Y_2_O_3_ (about 2–10 nm) was successfully synthesized by the improved wet chemical method. After that, spark plasma sintering (SPS) was employed to prepare yttria-dispersion-strengthened tungsten based alloys. The sintered W-Y_2_O_3_ alloy whose powder precursor was fabricated by improved wet chemical method possesses smaller grain size and higher relative density than the sintered W-Y_2_O_3_ alloy whose precursor fabricated by traditional wet chemical method. Moreover, the oxide nanoparticles are dispersed within tungsten grains (about 2–10 nm) and at tungsten grain boundaries more uniformly in the former one and its Vickers microhardness is up to 600 Hv.

## Experimental procedure

Yttria doped tungsten powder precursor with a nominal composition of W-5wt%Y_2_O_3_ was synthesized through a wet chemical process from (NH_4_)_10_[H_2_W_12_O_42_]·4H_2_O (APT) and yttrium nitrate hydrate Y(NO_3_)_3_·6H_2_O. 2 g of anion surfactant SDS and 2.57 g of yttrium nitrate hydrate were firstly dissolved in 120 ml of distilled water. After dispersing adequately under the condition of mechanical stirring and ultrasonic process, 20 g of APT was added into the solution. Then 20 ml of nitric acid (65–68%) was added into the solution for codeposition reaction. After 30 min, 140 ml of anhydrous ethanol was added into the formed suspension and left to react continuously for another 3 h. After that, the resulting suspension was filtered. The collected precipitate was washed with ethanol for four times to remove SDS and dried in vacuum drying oven at 60 °C for 24 h. Mechanical stirring and ultrasonic process were carried out throughout the whole process. The same procedure except SDS addition and ultrasonic process was used to synthesize another kind of W-Y_2_O_3_ composite powder.

The two kinds of as-obtained composite powder were calcined in a tube furnace, followed by reduction under pure hydrogen atmosphere. The calcination, 450 °C for 1 h, was carried out under argon atmosphere. However, the reduction was carried out in two steps, the first step at 600 °C for 3 h and the second step at 800 °C for 6 h. Finally, the powder was left in furnace to cool down to room temperature under hydrogen atmosphere.

A SPS machine “Dr. Sinter 1050” (Sumitoma coal mining company) was used to sinter these prepared powder precursors under vacuum condition. 10 g of each powder precursor was firstly pressed in a graphite die (12 mm in inner diameter) by manual. Then the die was placed into the SPS chamber. The temperature was firstly ramped to 600 °C and held for 2 min. Then the temperature was soared to 1600 °C and held for 2 min under a pressure of 50 Mpa. When the temperature was lower than 1500 °C, the heating rate was 100 °C/min. When the temperature was higher than 1500 °C, the rate was changed to 50 °C/min. The sintered alloy whose powder precursor was synthesized by traditional wet chemical method is denoted by sample 1 and the sintered alloy whose powder precursor was synthesized by improved wet chemical method is denoted by sample 2.

The phase composition and microstructure of the powder precursors and sintered alloys were examined by X-ray diffraction (XRD, D/MAX-2500) with Cu K*α* radiation, scanning electron microscopy (SEM, Hitachi Model No. S 4800) and transmission electron microscopy (TEM, JEM-2100) equipped with EDX detector, respectively. The density of the sintered alloys was measured by Archimedes method (in water at room temperature). The Vickers microhardnesses of the sintered alloys were tested on the polished surfaces under 200 gf load and a dwell time of 20 s at room temperature.

## Results and Discussion

**Figure 1:**
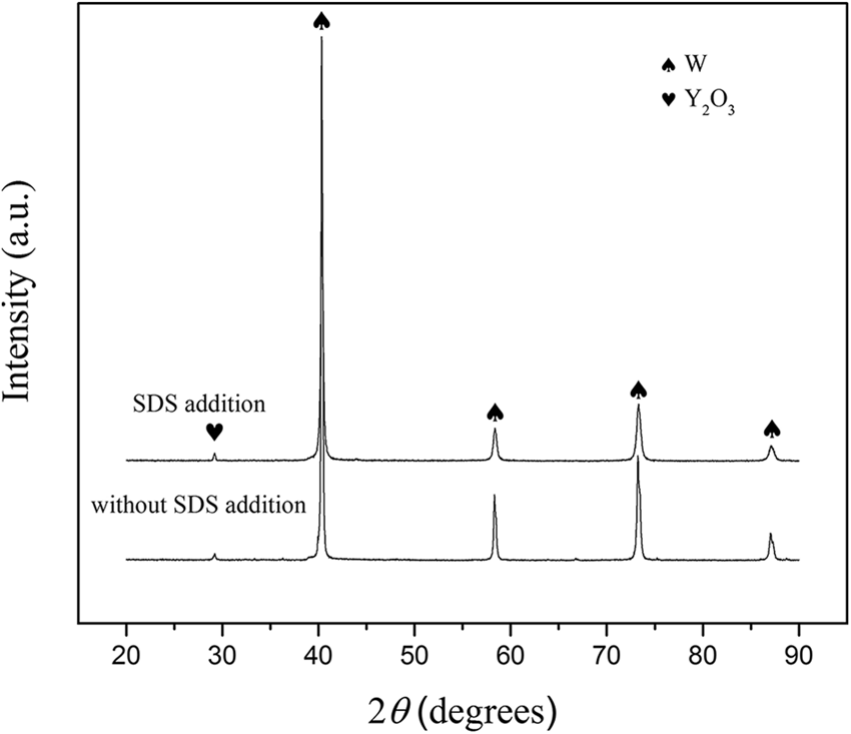
XRD patterns of the W-Y_2_O_3_ composite powder precursors fabricated by traditional and improved wet chemical method.

Figure 1 shows the XRD patterns of reduced W-Y_2_O_3_ composite powder precursors. Absence of other impurity phase peaks indicates that these composite powders have been reduced completely. Besides, it can be observed that the full width of half maximum (FWHM) of the peaks corresponding to tungsten become broader and the intensities of these peaks decrease after introducing SDS in traditional wet chemical method, which confirms the grain refining effect of SDS addition.

**Figure 2:**
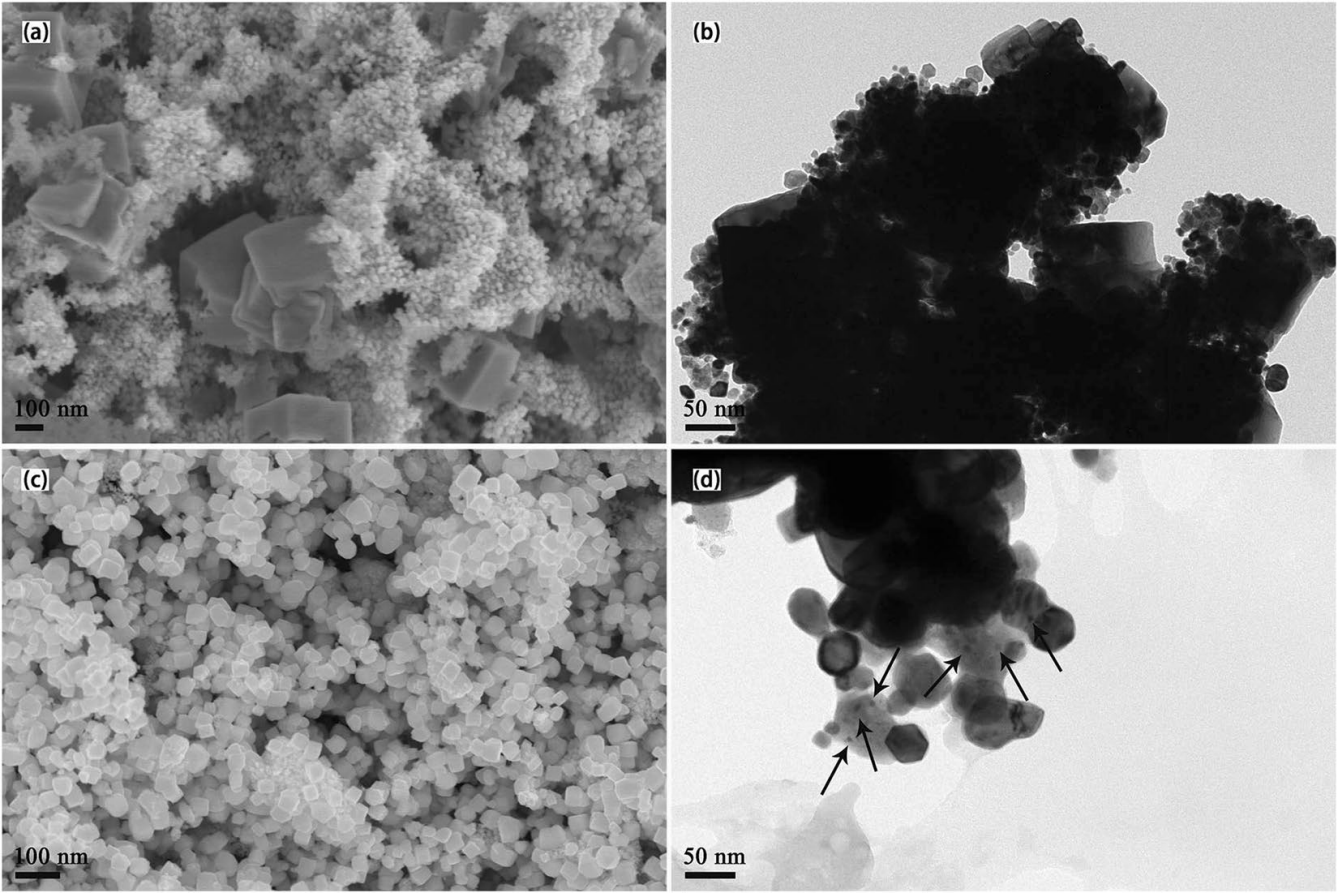
(**a**) SEM images of the reduced W-Y_2_O_3_ composite powder precursor fabricated by traditional wet chemical method, (**b**) TEM images of the reduced W-Y_2_O_3_ composite powder precursor fabricated by traditional wet chemical method, (**c**) SEM images of the reduced W-Y_2_O_3_ composite powder precursor fabricated by improved wet chemical method and (**d**) TEM images of the reduced W-Y_2_O_3_ composite powder precursor fabricated by improved wet chemical method.

**Figure 3:**
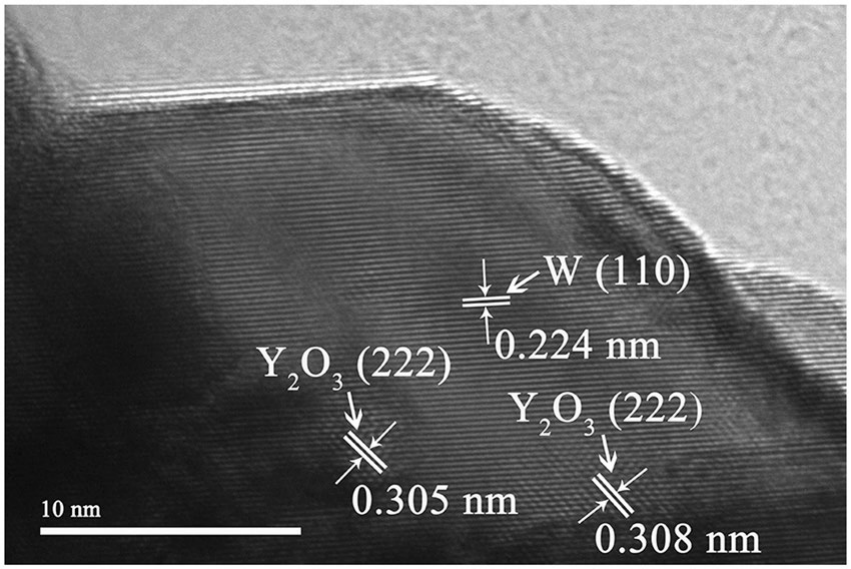
HRTEM image corresponding to a small region in Fig. 2d.

The SEM and TEM images of reduced W-Y_2_O_3_ composite powder precursors synthesized by improved and traditional wet chemical method are shown in Fig. 2. As seen from it, the powder precursor produced by traditional wet chemical method (Fig. 2a) consists of particles with two different morphologies. The larger particles with cubic shape have a size of 100–400 nm while the smaller particles (<50 nm) clustered into agglomerates present spherical shape. The TEM image corresponding to the powder precursor is shown in Fig. 2b and similar morphologies are also observed from it. The same bimodal size distribution in W-Y_2_O_3_ powder precursor was also observed by other researchers^19, 20^. Due to the huge specific surface area and large surface energy, the formed ultrafine tungstic acid crystal nucleuses have strong self-aggregation tendency, leading to grain growth during chemical reaction and then the appearance of bimodal size distribution after reduction. However, narrow size distributed tungsten grains are observed in the powder precursor synthesized by improved wet chemical method, as shown in Fig. 2c. Calculated using Scherrer’s formula, the average crystallite size of 39.6 nm was obtained for the composite powder precursor, which is in agreement well with the measured size from TEM (Fig. 2d). During the chemical reaction, the SDS adsorbed on the surfaces of tungstic acid crystal nucleuses will make them carry similar electric charges and then repel each other, hindering the crystal nucleuses from agglomeration and growth^23^. In addition, steric hindrance from SDS makes the grains dispersed, keeping good dispersibility. Thus, ultrafine grains with uniform size distribution can be obtained in the powder precursor in present work. Furthermore, for the powder precursor fabricated by improved wet chemical method, lots of nano-size particles (marked by black arrows) can be found within tungsten grains, as shown in Fig. 2d. In order to further confirm what these particles are, the high-resolution transmission electron microscopy (HR-TEM) image in the region was shown Fig. 3 and it is found that these particles are Y_2_O_3_. It is generally accepted that four oxygen atoms with negative charges on the polar head of SDS can interact with yttrium ions, forming complex precipitate that is mixed with APT uniformly^23^. When nitric acid is added in, the complex precipitate is dissolved and yttrium component exist mainly in the form of Y^3+^ ion. The liquid solution will promote the homogeneous mixing of the [H_2_W_12_O_42_]^10−^ and Y^3+^ ions and they will react and co-precipitate out^24^. In addition, co-deposition also can occur between the formed tungstic acid crystal nucleuses and Y^3+^ ions^25^, enclosing Y_2_O_3_ particles within W grains after reduction and forming a core-shell structure that is confirmed by the observation in Fig. 3. Isolated by tungsten matrix, these Y_2_O_3_ particles can hardly aggregate each other, keeping original size uniformly during subsequent SPS. Therefore, SDS, as anion surfactant, is highly beneficial to uniformity distribution of Y_2_O_3_ grains at tungsten grain boundaries and within tungsten grains, besides the refining effect on tungsten grains, as discussed above.

**Figure 4:**
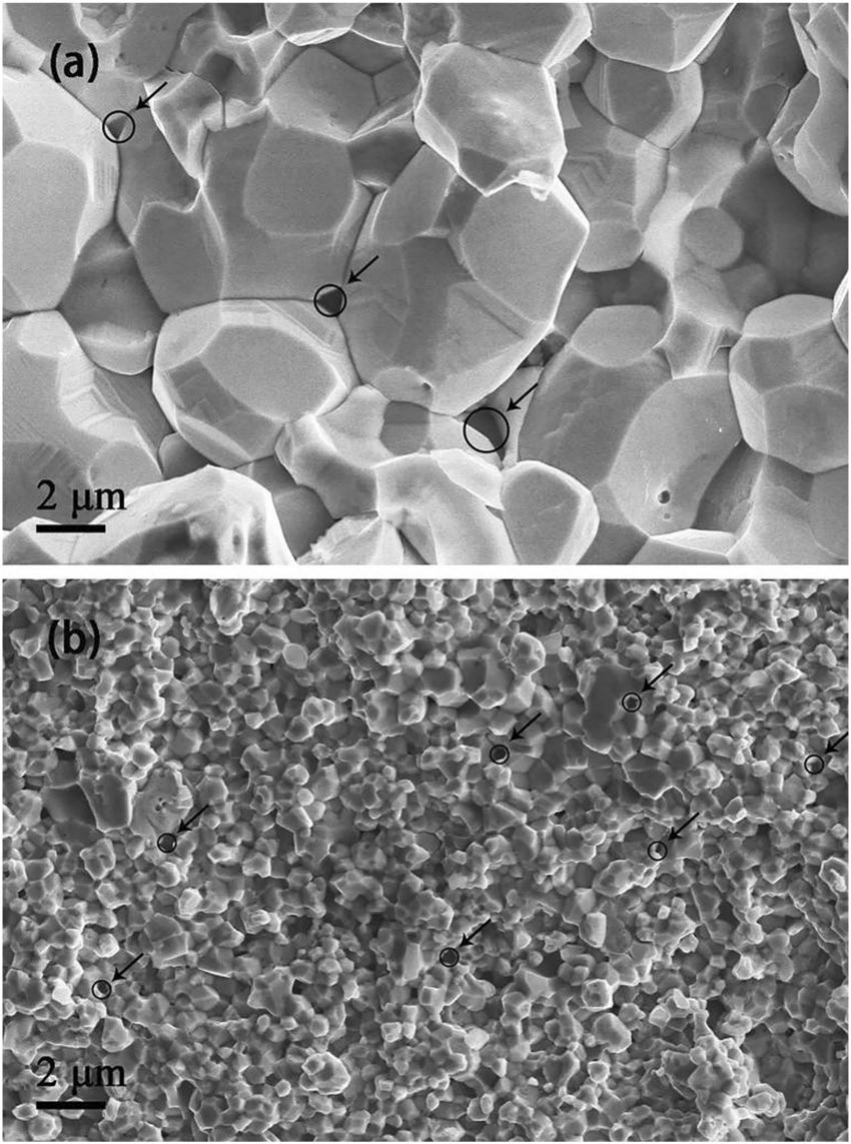
SEM images of the fracture surfaces (**a**) W-Y_2_O_3_ alloy fabricated by traditional wet chemical method and subsequent SPS, (**b**) W-Y_2_O_3_ alloy fabricated by improved wet chemical method and subsequent SPS.

The SEM images of sintered W-Y_2_O_3_ alloys with these two powder precursor are presented in Fig. 4. It is found that the average grain size of sample 1 and sample 2 are 5.82 and 0.76 μm, respectively. It means that the usage of powder precursor prepared by improved wet chemical method can lead to much more refined tungsten grains in the final sintered alloys than that prepared by traditional wet chemical method. From the SEM and TEM images (Fig. 2) and XRD patterns (Fig. 1) of the powder precursors, it is clear that the initial grain size of the powder precursor corresponding to sample 1 is much larger than that of sample 2. Besides, according to Ostwald ripening mechanism, the bimodal size distribution of tungsten grains observed in the powder precursor of sample 1 (Fig. 2a and b) will significantly lead to the abnormal grain growth during subsequent SPS. Compared with sample 1, the ultrafine and uniformly distributed Y_2_O_3_ grains in the powder precursor of sample 2 can hinder the grain boundary migration more effectively according to the Zener drag effect^24^, also contributing to the smaller W grain size. Therefore, based on the combined effect of these factors mentioned above, sample 2 possesses finer grains after SPS than sample 1. Till now, considerable research efforts have been devoted to prepare W-Y_2_O_3_ alloy with small grain size and high relative density simultaneously. However, a good balance between them is always difficult to be achieved in previous studies^17, 26^. With nearly full densification, the grain size of W-Y_2_O_3_ alloy fabricated by improved wet chemical method and subsequent SPS in our work is much smaller than that reported in previous studies.

**Figure 5:**
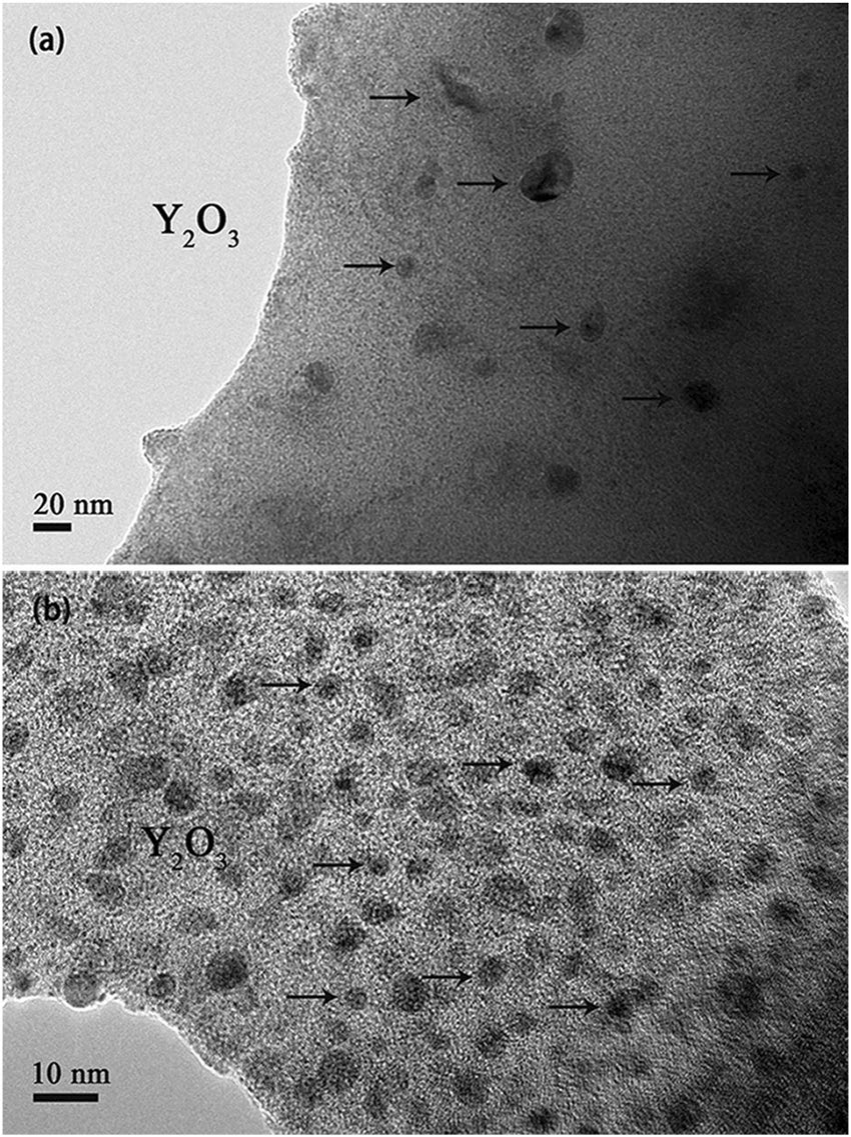
TEM images of the tungsten grain interior corresponding to W-Y_2_O_3_ alloy fabricated by (**a**) traditional wet chemical method and subsequent SPS and (**b**) improved wet chemical method and subsequent SPS.

Furthermore, many black particles (marked by black circles) distributed at grain boundaries can be observed for both samples in Fig. 4. Confirmed by the results of EDX spectrum, these particles are Y_2_O_3_ phase. One can also see that the Y_2_O_3_ particles in sample 1 are slightly larger than the ones in sample 2 and the size uniformity of the former is not so good as that of the latter, which is well in accord with the effect of SDS addition on the Y_2_O_3_ uniform distribution, as discussed above. To further investigate the detailed microscopic features within tungsten grains, the microstructures of both sintered alloys were characterized by TEM (Fig. 5). It is obvious that the size and distribution of Y_2_O_3_ particles (marked by black arrows) are significantly different for the two alloys. For sample 2 (Fig. 5b), Y_2_O_3_ particles with size from 2 to 10 nm are homogeneously dispersed within tungsten grains. Nevertheless, very few Y_2_O_3_ particles with size from 10 to 50 nm are found within tungsten grains of sample 1 (Fig. 5a). At the initial phase of SPS sintering, the W and Y_2_O_3_ grains are very small and the velocity of W grain boundary migration is larger than the speed of Y_2_O_3_ particles movement because of high driving force. Then, the grain boundaries will cross partial Y_2_O_3_ particles dispersed at W grain boundaries so that form the intragranular structure except that formed in powder precursor (Fig. 3). With tungsten grains growing up, the driving force for grain boundary migration decreases and is close to the pinning force provided by Y_2_O_3_ particles, resulting in these Y_2_O_3_ particles still staying at grain boundaries^27, 28^. Besides, due to the aggregation and coarsening effects during SPS^24^, these Y_2_O_3_ particles will diffuse, aggregate and grow up. Therefore, the smaller initial W and Y_2_O_3_ grains are, the more Y_2_O_3_ particles distributed at grain boundaries will be enclosed within W grains during subsequent SPS. Based on the analysis, it is understandable that only a few Y_2_O_3_ dispersoids with size of approximate 10–50 nm distribute unevenly within tungsten grains of sample 1 and the majority of them distribute at grain boundaries when comparing with sample 2.

**Figure 6:**
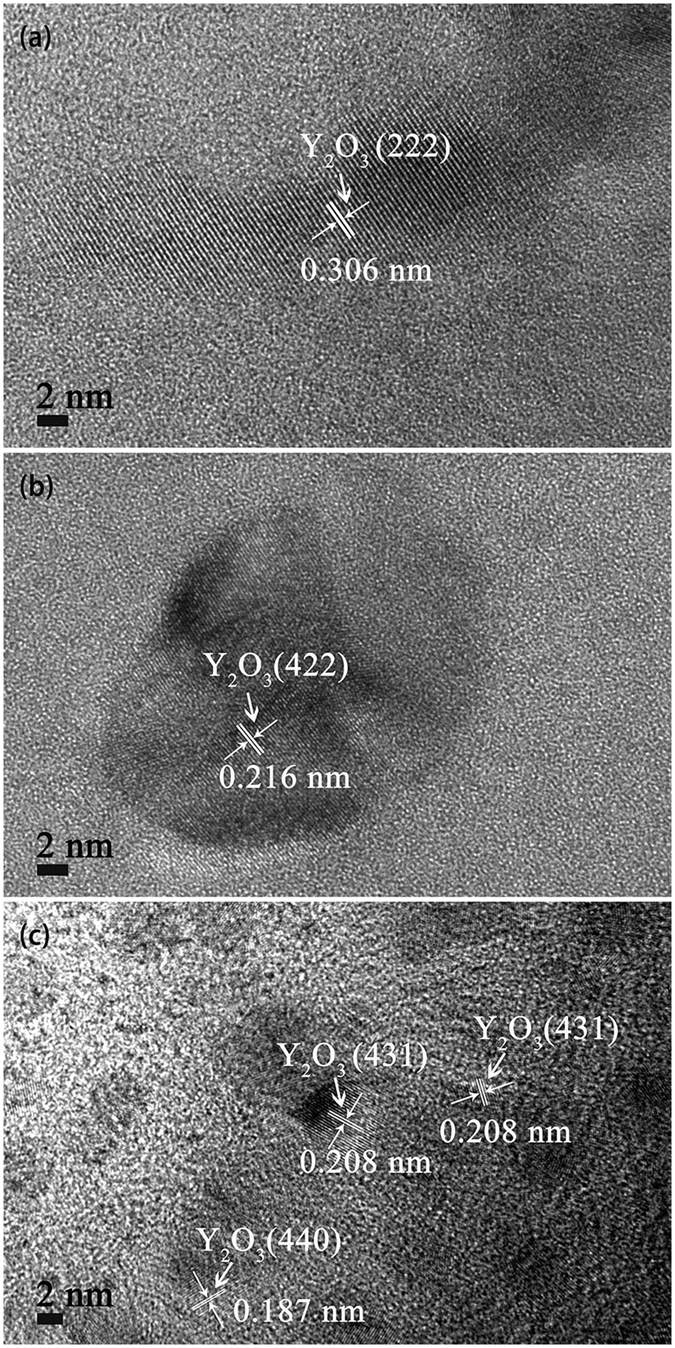
HRTEM images of Y_2_O_3_ particles within tungsten grain of W-Y_2_O_3_ alloy fabricated by (**a**) and (**b**) traditional wet chemical method and subsequent SPS, (**c**) improved wet chemical method and subsequent SPS.

The HRTEM images of Y_2_O_3_ particles within tungsten grains for both samples are shown in Fig. 6. Different from the near spherical Y_2_O_3_ particles within tungsten grains of sample 2 (Fig. 6c), the Y_2_O_3_ particles within tungsten grains of sample 1 (Fig. 6a and b) have various shapes. For the powder precursor produced by improved wet chemical method, due to the highly homogeneous distribution of yttrium component under the action of SDS, the adjacent yttrium component will aggregate, crystallize and then form spherical Y_2_O_3_ particles during calcination. However, without SDS addition, the inhomogeneous distribution of yttrium component will result in larger Y_2_O_3_ particles with various shapes. At the same time, the distance of Y_2_O_3_ particles moving is restricted by low temperature of thermal processing (calcination and reduction) and W matrix, resulting in nano-sized Y_2_O_3_ particles after reduction. During the process of SPS, grain boundary migration makes partial Y_2_O_3_ particles at grain boundaries enclosed within W grains for the two samples, as described above. Compared with spherical Y_2_O_3_ particles, the particles with other shapes have higher surface energy and tend to spheroidize spontaneously during SPS according to the principle of minimum energy. Nevertheless, the remarkable features of SPS, fast heating rate and short sintering time, restrict the spherical conversion. So, a conclusion can be drawn that SDS addition also can remarkably control the size, shape and uniformity distribution of Y_2_O_3_ particles within tungsten grains.

The relative density, grain size and Vickers microhardness of the sintered W-Y_2_O_3_ alloys are listed in Table 1. The relative density of sample 1 and 2 is 96.28% and 99.00%, respectively. There are several reasons behind the density difference between these two samples. On the one hand, with smaller grain size and narrower size distribution, the powder precursor corresponding to sample 2 possesses higher sintering activity than the powder precursor of sample 1, resulting in this kind of powder precursor being consolidated more easily at the same sintering condition in the subsequent SPS process. On the other hand, compared with sample 1, a more uniform Y_2_O_3_ distribution in the powder precursor of sample 2 is in favor of sintering densification. As listed in Table 1, the Vickers microhardness of sample 2 was measured to be 598.74 Hv, much higher than that of sample 1. The higher Vickers microhardness originates from finer grains and higher relative density, as shown in Table 1. Besides, large quantities of Y_2_O_3_ particles within W grains of sample 2 increase the mean projection area that is proportional to intersection probability between dislocations and Y_2_O_3_ particles^29^. Thus, compared with sample 1, the oxide particles uniformly dispersed within W grains of sample 2 can generate, pin down and accumulate dislocations within W grains more effectively, contributing to the increase of microhardness significantly. A hardness value of about 393 Hv was obtained by sintering W-1.15 wt%Y_2_O_3_ powder precursor at same temperature for 3 min, which results from the larger grain size (7.07 μm)^19^. When the same relative density (99%) was obtained by sintering the W-5wt%Y_2_O_3_ powder precursor prepared by mechanical alloying, growth of tungsten grain (3.7 μm) makes the hardness value of 525 Hv lower than that in this paper^17^. In addition, for the W-Y_2_O_3_ powder precursor fabricated by sol-gel with PEG addition, the sintered alloy possesses a hardness value of about 600 Hv. However, the relative density of the sintered alloy is only 93.8%^22^. Compared with these previous results, our sample fabricated by improved wet chemical method and subsequent SPS possesses not only high microhardness but also ultrafine grains and high relative density.

**Table 1:** Relative density, grain size and Vickers microhardness of the sintered W-Y_2_O_3_ alloys.

Samples	Relative density(%)	Grain size(μm)	Hv
Sample 1	96.28	5.82	354.92
Sample 2	99.00	0.76	598.74

## Conclusions

Yttria-dispersion-strengthened tungsten based alloy was fabricated by an improved wet chemical method where anion surfactant SDS was introduced and subsequent SPS. For comparison, the W-Y_2_O_3_ alloy was also produced by traditional wet chemical method and subsequent SPS. It is found that SDS addition has a significantly positive effect on the size uniformity and refinement of tungsten grain as well as the homogeneous distribution of Y_2_O_3_ particles. This high-quality W-Y_2_O_3_ composite powder can serve as good precursor for the sintering of high-performance yttria-dispersion-strengthened tungsten based alloys. For the alloy whose powder precursor was produced by improved wet chemical method, the microstructure characterization shows that the Y_2_O_3_ particles are dispersed both within tungsten grains (2–10 nm) and at the tungsten grain boundaries homogeneously. However, the majority of Y_2_O_3_ particles in the form of large agglomerations are dispersed at grain boundaries and only a few Y_2_O_3_ particles with size of 10–50 nm are distributed within tungsten grains non-uniformly in the W-Y_2_O_3_ alloy whose powder precursor was fabricated by traditional wet chemical method. Moreover, the W-Y_2_O_3_ alloy prepared by improved wet chemical method and subsequent SPS possesses the smaller grain size (0.76 μm) and the higher relative density (99.00%) than that produced by traditional wet chemical method and subsequent SPS. Besides, its Vickers microhardness is up to 600 Hv. All these results suggest that the introduction of SDS surfactant into traditional wet chemical method is a promising way to obtain high quality composite powder precursor for fabricating high performance oxide-dispersion-strengthened tungsten based alloys.
